# Platform switch hybrid zygoma implants improve prosthetics and marginal bone protection after extra‐sinus placement

**DOI:** 10.1111/cid.12878

**Published:** 2020-02-11

**Authors:** Paweł Aleksandrowicz, Marta Kusa‐Podkańska, Witold Tomkiewicz, Lidia Kotuła, Jan Perek, Joanna Wysokińska‐Miszczuk

**Affiliations:** ^1^ Department of Periodontology Medical University of Lublin Lublin Poland; ^2^ Departament of Oral Surgery Medical University of Warsaw Warsaw Poland; ^3^ Department of Clinical Genetics Medical University of Lublin Lublin Poland

**Keywords:** edentulous atrophic maxilla, extra‐sinus, platform switch, zygoma implants

## Abstract

**Purpose:**

The aim of our studdy is clinical evaluation of Platform switch hybrid zygoma implants.

**Materials and Methods:**

117 zygomatic implants were followed up during this time. They included 55 Brånemark System zygoma implants, 38 Noris implants, and 24 novel iRES hybrid implants with platform switch.

**Results:**

Bone quality and quantity are the prerequisite for successful implant treatment. Zygomatic implants are intended for patients with severely resorbed maxilla that cannot accommodate conventional implants without prior extensive bone grafting. Such regenerative procedures, like sinus lifts, prolong implant rehabilitation to several months (12–18). Furthermore, extensive grafts are less predictable showing varying degrees of graft resorption. Zygoma implants enable full, often immediate, reconstruction of the upper dental arch without the need for sinus lift treatment. The original zygoma protocol runs the implants through the sinus, requires general anesthesia, and positions the prosthetic platform of the implants on the palate, which makes prosthesis cumbersome. It also induces risk for post‐op sinusitis. Extra‐sinus approach with novel zygoma hybrid implants bypasses sinuses and positions the implant prosthetic platform on the crest allowing for same good prosthetics as on conventional dental implants. Furthermore, crestal threads and a platform‐switch, of the novel zygoma design, increase implant anchorage and minimize marginal bone loss. The study presents evolution of zygoma implant rehabilitation protocol and zygoma implant design in our clinical practice over 15 years (2004‐2019).

**Conclusion:**

Extra‐sinus zygomatic implant placement lowers the risk of post‐op sinusitis and makes procedure possible to be done in local anesthesia.

## 1 INTRODUCTION

Loss of teeth leads to bone atrophy of the alveolar crest^1^, ^2^, ^3^, ^4^, ^5^, ^6^, ^7^, ^8^, ^9^ up to 1/3 of the original height within a few weeks after extraction. In the following years, atrophy progresses both from the crest and the sinus as a result of invasive proliferation of the maxillary sinus mucosa.^10^

The shape and structure of the zygomatic bones presented good anchorage alternative for longer implants (zygomatic implants). The efficacy of rehabilitation with zygomatic implants in maxilla is well documented.^11^, ^12^, ^13^ The limitations for the more comprehensive use of this method were invasive surgery under general anesthesia and prosthetic challenges with palatally positioned implant heads.

This study presents evolution of the protocol from intrasinus in general anesthesia into extra‐sinus in local anesthesia^14^ and from palatal to crestal position of the implant heads for easier prosthetics. These changes required a new implant design: hybrid surface with crestal threads and platform‐switch internal connection for better anchorage and marginal bone care.

**Figure 1:**
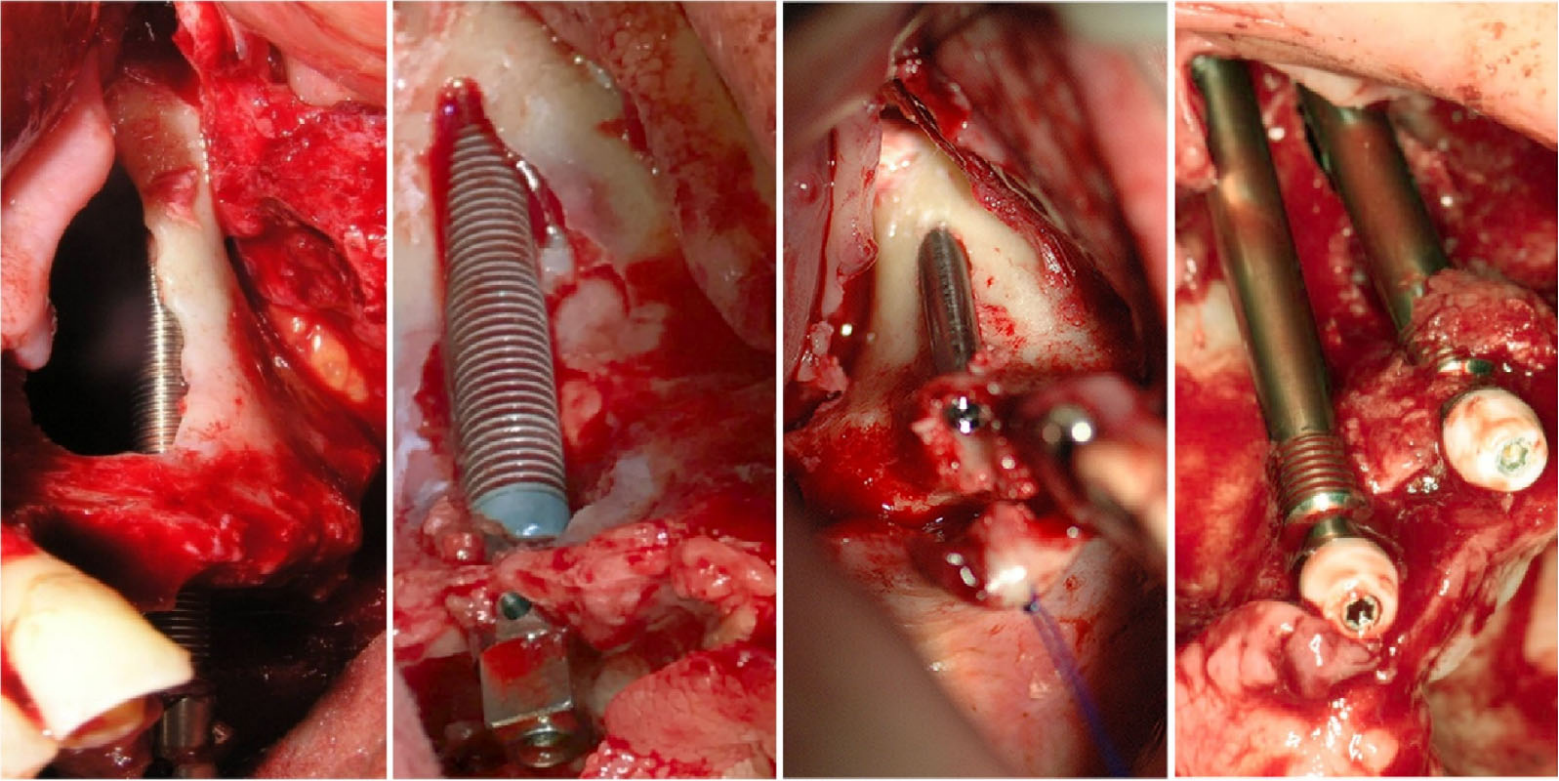
Evolution of zygoma implants and surgical protocol

Zygomatic implants first introduced by professor Per‐Ingvar Brånemark in 1988^3^ had machined surface and a lengths from 35 to 52.5 mm. The original protocol was two zygoma implants placed bilaterally (one on each side) and four regular implants in the anterior maxilla. Zygomatic implants ran through the lumen of the maxillary sinus, with the implant heads sticking out on the palatal side of the alveolar crest^15^ (Figure 1).

Zygoma implants reduced overall treatment (full upper arch rehabilitation) time and eliminated the need for bone grafting into maxillary sinus.^16^, ^17^ The protocol was then modified to four zygomatic implants two on each side^18^ for patients who do not have enough bone in the front of maxilla.

The goal of our clinical research was first to facilitate prosthetics by moving the implant heads to the crestal ridge. Therefore, we began to place zygoma implants more mesially, in the front of maxilla.^19^ The 30 mm implants went through the sinus cavity and the implant heads sticked out at the second molar site. Prosthetics became normal then and did not require any additional prosthetic elements towards the palate.

Then we wanted to bypass the sinus to make procedure less invasive and minimize the risk of post‐op sinusitis. So we went with drills more buccally that the implant does not pass through the maxillary sinus but runs in the sinus wall or outside. Crestally we wanted to preserve a bony bridge around implant head as much as possible to prevent soft tissue recession around the prosthetic abutment. We used implants 40 or 45 mm^20^, ^21^ long. The implant head was at the position of second premolar or first molar but not exactly on the crest. In this protocol, however, prosthetic framework had to be thicker and palatal extensions were often necessary.

**Figure 2:**
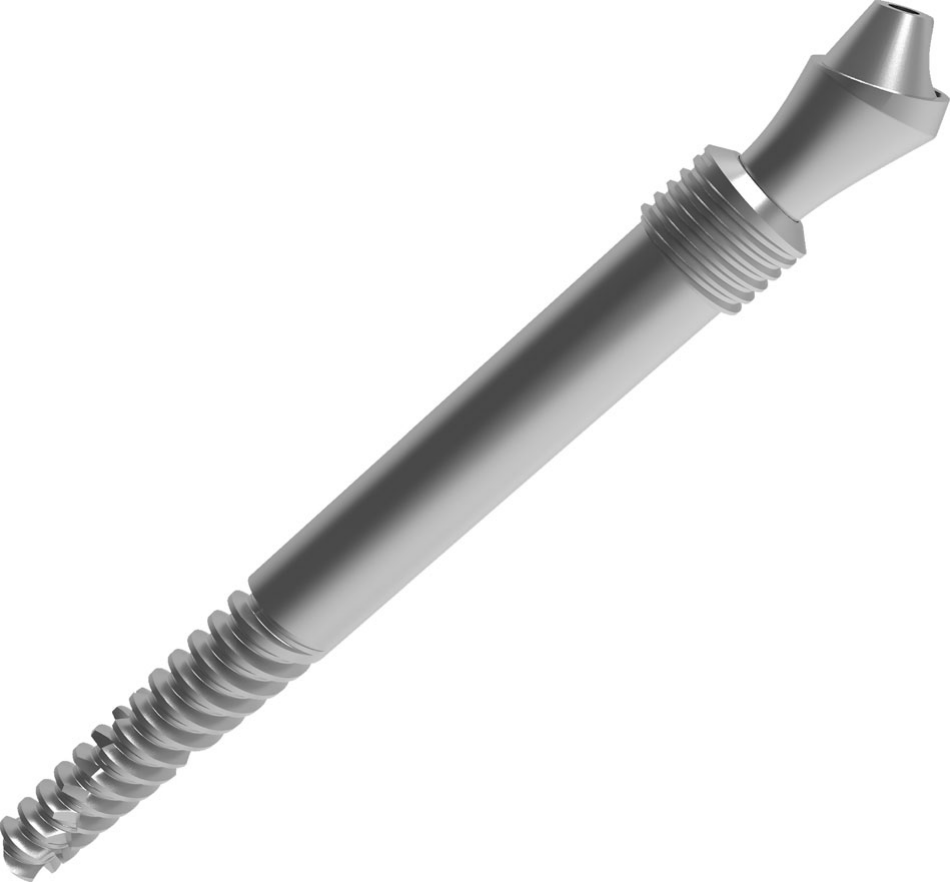
Patient with advanced periodontal disease rehabilitated with Noris zygomatic implants—x‐rays before and after surgery. Extraoral images at 1 year follow up—visible

**Figure 3:**
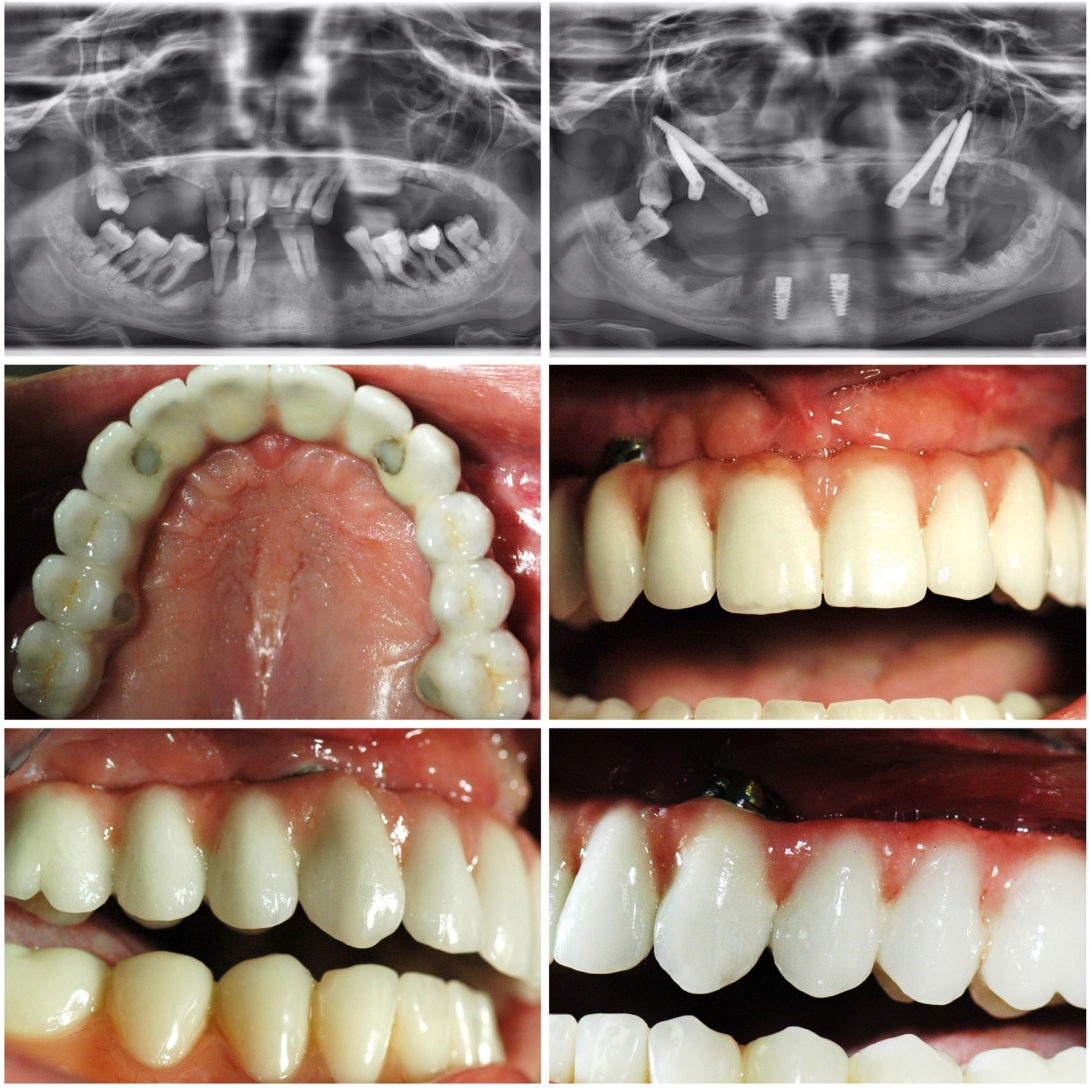
Zygomatic hybrid implant with “platform‐switch” prosthetic connection

Further evolution of the protocol for extra‐sinus placement considered improvement of abutment position and also how to avoid mucosal recession around it (Figure 2). We needed an implant with crestal thread and internal platform switch connection so the implant could be placed below the crest (subcrestal placement) with prosthetic abutment emerging on the top of the alveolar crest (Figure 3).

**Figure 4:**
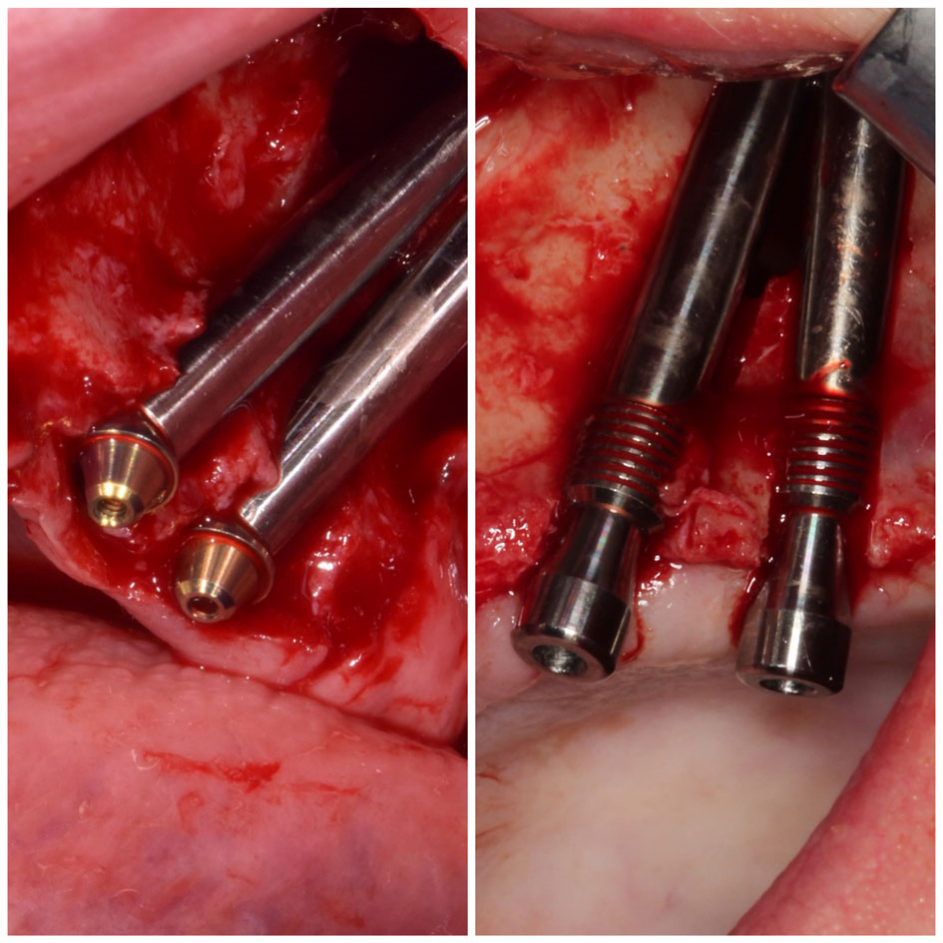
Two types of zygomatic implants classic left—(Noris Medical) and right—hybrid with platform‐switch (iRES)

The novel implant is a hybrid with rough (sand blasted and double attached) surface at the intra‐zygomatic apex, machined surface at nonthreaded central part (in contact with the maxillary sinus wall or cavity), and crestal threads to minimize periimplantitis risk there. Implants are 30 to 65 mm long and are adapted to the surgical protocol with Le Fort I simultaneous osteotomy. The Multi‐Unit abutments have a “fleur‐de‐lys” emerging profile with platform‐switch for both bone and soft tissues (Figure 4).

The hybrid implant's surgical protocol involves the extra‐sinus implant placement in the zygomatic bone body. The hybrid implant needs to be placed subcrestally in order to position the abutment on the top of the alveolar crest.

**Figure 5:**
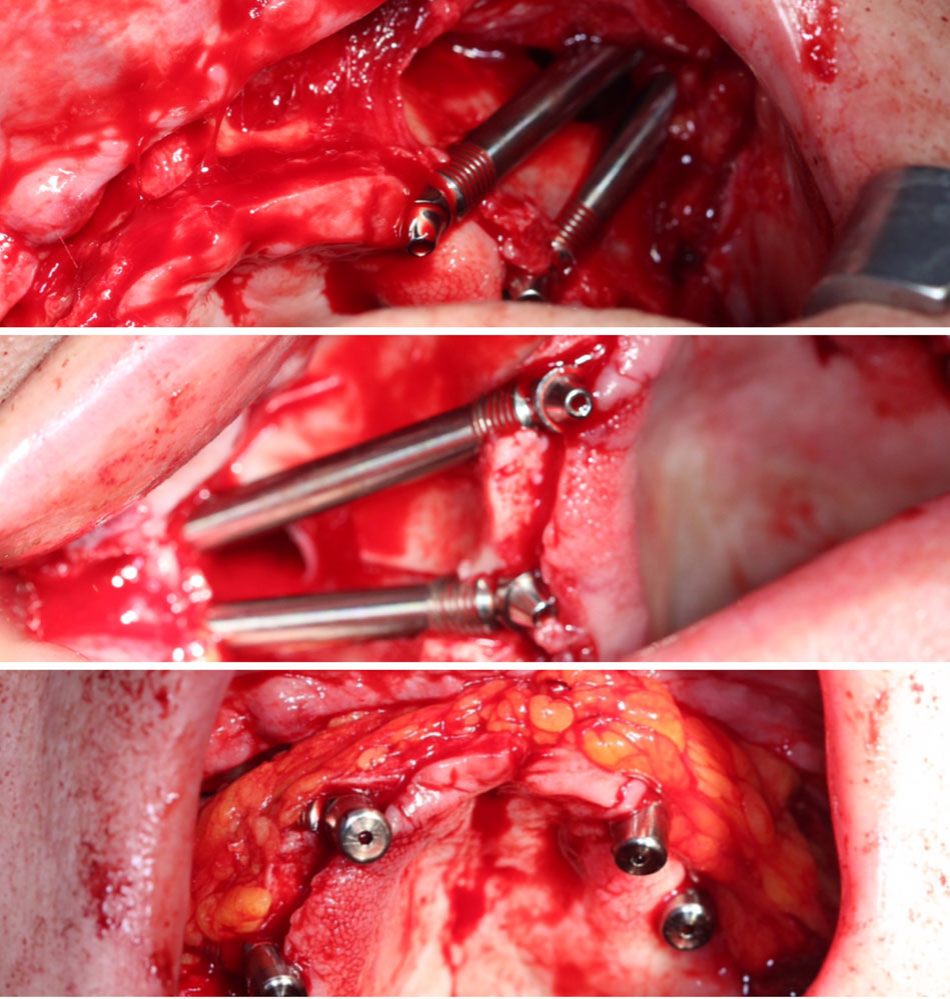
Augmentation of soft tissues with fat pads Corpus adiposum buccae

Fat pads soft tissue augmentation. In patients with a thin mucosal biotype we did soft tissue augmentation with pedunculated Bichata/Corpus adiposum buccae/fat pads (Figure 5) to avoid mucosal recession around the abutment.

## 2 ANESTHESIA

The extra‐sinus zygoma implants were done in local anesthesia both intra‐oral and extra‐oral, percutaneously in the zygomatic bone area to detach the periosteum for subsequent preparation of the mucoperiosteal flap as well as detachment of the muscle *m. zygomaticus major et minor* attachment.

We also performed the procedure under general anesthesia at the patient's request.

After a thorough physical examination, we qualify the patient for surgery according to the ASA scale. Due to the extent of the procedure and its duration, we use general anesthesia in a complex manner—intravenously and intratracheally. We collect a patient's consent each time after routine preanesthetic testing and risk assessment. We place the patient in a prone position—with the option of using the Trendelenburg position. Then we use standard vital functions monitoring, that is, automatic periodic RR measurement, ECG recording from four precordial leads, pulse, and arterial blood saturation recording. We perform venipuncture with a 1.4 mm Venflon cannula and intravenous induction: Fentanyl 0.002 mg/kg + Norcuron 0.07 mg/kg + Thiopental 3.45 mg/kg—using passive oxygenation with 100% oxygen at the same time. Switching to active oxygenation—after muscle relaxation—we perform atraumatic tracheal intubation through the nose—a 7 mm diameter profiled pulmonary silicone tube with a low‐pressure sealing cuff. After establishing the artificial respiration and starting ventilation in CMV mode with 100% oxygen, we change the breathing mixture to 67% nitrous oxide and 33% oxygen using a standard anesthesia fan—for example, Fabius‐Draeger with full control of ventilation parameters. We use Fentanyl—0.0005 mg/kg/h to carry out anesthesia, Norcuron 0.01 mg/kg/h for relaxation and isotonic fluids/PWE/2.5 mL/kg/h. After the procedure, there is a transition to 100% oxygen and spontaneous breathing. Then, after achieving full contact and recovery of the patient's muscular strength, we carry out extubation. After the procedure, we apply postoperative analgesia with an automatic syringe Fentanyl 0.0006 mg/kg/h for 24 hours with a positive result according to the subjective pain scale.

## 3 MATERIALS AND METHODS

The study involved 29 women and 20 men aged 33 to 81 who were treated at the Department of Periodontology of the Medical University in Lublin. Patients were qualified for surgery by one doctor after ENT consultation. Among the patients 16 were treated for hypertension and six were smokers.

Each patient had an OPG and CBCT scans done for optimal diagnostics. The first patient in the study group received zygomatic implants in 2004 and the last patient in 2019. The cumulative follow up was 180 months.

The study protocol was positively evaluated by the local Bioethics Committees at the Medical University of Lublin on day January 31, 2019 (number resolution KE‐0254/43/2019).

## 4 RESULTS

Three types of zygomatic implants were used. Total 117 implants. Zygomatic implants (a), with a fully sandblasted and acid‐etched surface, accounted for 47% (n = 55). The group (B) of implants were smooth with rough threaded apex—32% (n = 38).

Hybrid implants (C), with rough threaded apex and machined (nonthreaded) body and crestal threads accounted for 21% (n = 24).

General anesthesia was done to patients who received an intra‐sinus implant—35.04%. All extra‐sinus procedures were performed under local anesthesia—64.96% (Table 1).

**Table 1:** Position of the implant in relation to the maxillary sinus

Position of the implant (1—in the sinus lumen; 2—extra‐sinus)	A (Group: zygomatic implants)		B (Group: Noris implants)		C (Group: hybrid implants)	
	N	%	N	%	N	%
1	40	72.73	1	2.63	0	0.00
2	15	27.27	37	97.37	24	100.00

*Note*: “1″—N = 41 (35.04%); “2″—N = 76 (64.96%).

The crestal position of the prosthetic abutments was achieved in 71.79% (Table 2).

**Table 2:** Position of the implant head/prosthetic abutment: on the crest or palatally

Placement of the implant (1—top of the crest; 2—slightly palatal)	A (Group: zygomatic implants)		B (Group: Noris implants)		C (Group: hybrid implants)	
	N	%	N	%	N	%
1	23	41.82	37	97.37	24	100.00
2	32	58.18	1	2.63	0	0.00

*Note*: “1″—N = 84 (71.79%); “2”—N = 33 (28.21%).

The implants used in the study were from 30 to 50 mm long. The most commonly implanted zygomatic implants were 45 mm—32.48% (Table 3).

**Table 3:** Implant lengths

Length of zygomatic implant	A (Group: Zygomatic implants)		B (Group: Noris implants)		C (Group: Hybrid implants)	
	N	%	N	%	N	%
30	15	27.27	0	0.00	0	0.00
35	6	10.91	2	5.26	0	0.00
40	7	12.73	14	36.84	10	41.68
45	18	32.73	14	36.84	6	25.00
47.5	6	10.91	5	13.17	4	16.66
50	3	5.45	3	7.89	4	16.66

During follow‐up visits, periodontal examination with a calibrated plastic tube, periimplantitis was found in around 3% of classic zygomatic implants (Table 4) and maxillary sinusitis was below 6% (Table 5). The immediate loading was applied in 22 patients. Zygomatic implants in remaining 27 patients were loaded within 3‐6 months after surgery.

**Table 4:** Periimplantitis in zygomatic implants

Percentage of periimplantitis around zygoma	A (Group: zygomatic implants)		B (Group: Noris implants)		C (Group: hybrid implants)	
	N	%	N	%	N	%
0	52	94.55	38	100.00	24	100.00
1	3	5.45	0	0.00	0	0.00

*Note*: “0”—N = 114 (97.44%); “1”—N = 3 (2.56%).

**Table 5:** Post‐op sinusitis

Percentage of sinusitis on zygomatic implants (0—no sinusitis; 1—sinusitis)	A (Group: zygomatic implants)		B (Group: Noris implants)		C (Group: hybrid implants)	
	N	%	N	%	N	%
0	51	92.73	36	94.74	23	95.83
1	4	7.27	2	5.26	1	4.17

*Note*: “0”—N = 110 (94.02%); “1”—N = 7 (5.98%).

In the study group, about six implants (11%) Brånemark System and six Noris implants (16%) were exposed. There was no mucosal recession around the prosthetic abutments of hybrid implants.

## 5 DISCUSSION

The original Brånemark technique of placing zygomatic implants in patients with severely resorbed maxilla opened new opportunities for predictable and even immediate rehabilitation of such patients without grafting the sinus. With time, however, intra‐sinus placement of completely threaded and rough implants, palatal location of implant heads and general anesthesia were the limiting factors, for making zygoma treatment more common, due to the risk of post‐op sinusitis, implant failures, prosthetic challenges.

Therefore, extra‐sinus placement of hybrid (rough/machined) surfaced implants with crestal threads and internal platform switch connection lowers the risk of sinusitis and implant failure. Furthermore, subcrestal placement and platform‐switched abutments on the crest—make prosthetics more comfortable for the patient and predictable as with conventional dental implants.^22^

The novel implant design reduces gingival recession around prosthetic abutments due to platform‐switch applied.^23^, ^24^

Furthermore the results of our study indicate efficacy of immediate loading of extra‐sinus zygomatic implants which is the major benefit for the patient and treating team.^25^ Prosthetic loading 3‐6 months after surgery is also very popular among authors doing similar research. The overall failure rate of zygomatic implants in our study does not differ from the reported by other authors and amounts to 1.7%.^26^

According to our knowledge, no report on zygoma platform switch hybrid implants placed extra‐sinus has been published yet and therefore our findings may be encouraging for other investigators to further examine and popularize this graft‐less method of full and frequently immediate rehabilitation of highly compromised patients.

## 6 CONCLUSION

Extra‐sinus zygomatic implant placement lowers the risk of post‐op sinusitis and makes procedure possible to be done in local anesthesia. The use of hybrid implants lowers the risk of periimplntitis, sinusitis and implant failure. Crestal threads and internal platform‐switch connection enable subcrestal placement and on‐crest emerging of prosthetic abutment hence making prosthetics as good as on conventional dental implants. Soft tissue augmentation with fat‐pads can be made in patients with a thin soft tissue biotype to avoid gingival recession. The overall failure rate of zygomatic implants in our study does not differ from the reported by other authors and amounts to 1.7%.^26^

The use of zygomatic implants is often a rescue procedure after complications in patients who have previously received conventional implant treatment.

## CONFLICT OF INTEREST

The authors declare no conflicts of interest.
